# SegmentGeometry: A Tool for Measuring Second Moment of Area in 3D Slicer

**DOI:** 10.1093/iob/obac009

**Published:** 2022-02-28

**Authors:** Jonathan M Huie, Adam P Summers, Sandy M Kawano

**Affiliations:** Department of Biological Sciences, George Washington University, Washington, DC 20052, USA; Biology and SAFS, Friday Harbor Laboratories, University of Washington, Friday Harbor, WA 98250, USA; Department of Biological Sciences, George Washington University, Washington, DC 20052, USA

## Abstract

Second moment of area is a measure of how well the cross-section of a beam will resist bending because of its shape. Many have used second moment of area to investigate the mechanical adaptations of biological structures from stingray jaws to animal limb bones. In this context it is important to acknowledge the assumptions of beam theory, in which second moment of area plays a key role, if reasonable results are desired. For example, to minimize shear the structure should be at least 10 times longer than it is wide and deflection should be minimal. Analyzing the internal geometry of biological structures has never been easier or more accessible given the wide, and growing availability of micro-CT scans. Here, we offer a guide on the care that needs to be taken when interpreting second moment of area, and present open-access, open-source software that can process hundreds if not thousands of structures in a short time frame. *SegmentGeometry*, an extension for the open-source imaging platform 3D Slicer, iterates slice-by-slice through 3D structures to calculate second moment of area and other cross-sectional properties. We analyzed 2 case studies to demonstrate the power of this tool and to highlight interpretations that can be gleaned from second moment of area. Second moment of area is just one part of the Euler–Bernoulli beam theory and considering the full equation would greatly increase the number and diversity of questions that can be answered.

## Introduction

In 1750 Euler and Bernoulli formalized what has come to be known as classical beam theory (Truesdell and Euler 1960). They proposed a formula that explained the deflection of a long aspect ratio beam under load. Biologists focus on two forms of this equation. The first is a cantilever beam with an axial point load at the free end, which can model flexion in the radial bone when a weight is held in the hand, or flexion in the jaw when prey contacts a tooth at the tip of the jaw (Eq. 1). The second form of the equation is a simply supported beam, a long element supported at both ends and point loaded in the middle. This form is often used to extract material property data from a three point bending test (Eq. 2). In either case, Euler-Bernoulli's formula is simplified to an equation where the deflection (ẟ) depends on a scalar coefficient multiplied by the length (*L*) of the beam cubed, the force of loading (*F*), the inverse of the elastic or Young's modulus (*E*), and the inverse of the structural descriptor, second moment of area (*I*). Other cases of the deflection equation, for example, distributed loads, end moments, and compound loading, lead to similar equations with different scalar coefficients. Classical beam theory has been used in many biological applications that include, but are not limited to, inferring bipedalism in hominins, determining where in a stingray's jaws a hard prey item might be cracked, comparing the limb adaptations of ecologically diverse mammals, investigating the biomechanical diversity of extinct crocodylomorph jaws, and identifying the response of calcified algae to ocean acidification (Brassey et al. 2013; Stubbs et al. 2013; Newcomb et al. 2015; Doube et al. 2018; Kilbourne and Hutchinson 2019; Marchi et al. 2019; Rutledge et al. 2019).

(1) \begin{equation*}\delta = \frac{{F{L^3}}}{{3EI}}\ \end{equation*}

(2) \begin{equation*}\delta = \frac{{F{L^3}}}{{48EI}}\ \end{equation*}

The beauty of the relationship lies in the breadth of the variables, with their phylogenetic variability and experimental approachability. In diverse experimental systems, a subset of the variables can be quantified empirically, and the rest can be deduced either by solving the equation or by applying heuristics to set boundaries on the values. The ease of use and wide applicability of beam theory to a diverse range of structures has made it a popular mechanism to assess the structural integrity of biological structures, but some variables are more easily quantified than others. For instance, the second moment of area represents how well the beam will resist bending based on its shape, and can be easily and quickly measured with low cost equipment and minimal procedural knowledge. However, there are certain assumptions that must be met for beam theory to provide reasonable results. Here, we propose to clarify the conditions under which the formula applies, provide guidelines for the care that should be taken when defining anatomical planes and axes, and develop a new workflow that minimizes opportunities for error and violating model assumptions while being streamlined enough to process tens to thousands of specimens in a short period of time.

First, we will outline the assumptions of Eq. 1 and Eq. 2 and then proceed to address the pitfalls associated with each term in the equation. There are two foundational assumptions: (1) the deflections are small and (2) shear plays no role in the deformation of the beam. The first assumption is typically reported in mechanics textbooks as being deflections that are less than 10% of the length of the beam. This is not an issue with most load bearing skeletal elements; however, some long thin bones—bat phalanges and bird hyoids, for example—deform far more than this (Swartz et al. 1992; Jung et al. 2016). The issue of shear is one of degree: it is almost impossible to load a skeleton purely in bending, but the effects of shear decrease the longer and more slender the element is. Empirically, an element with a length to thickness ratio of at least 10:1 or higher will help reduce the shear component to a negligible contributor to deflection (Martin and Boardman 1993; Horton and Summers 2009; Kourtis et al. 2014).

The numerator of Eq. 1 and Eq. 2 incorporates information about the size of the beam and the loads that are applied to it. The length of a beam (*L*) can be surprisingly tricky to measure. Some beams are curved, others have processes that extend beyond the pivot point, and others are composed of radically different materials (i.e., cartilaginous versus calcified regions in a long bone), which poses a challenge for deciding where to set the endpoints of the beam (Milne 2016; Molnar 2021). The force (*F*) applied to a beam can sometimes be measured, either as external forces (e.g., ground reaction forces) with a force plate or as internal forces (e.g., muscle forces) with a strain gauge on a tendon (Biewener et al. 1988). However, incorporating *in vivo* loading regimes can be challenging because of time-dependent factors (Ker et al. 1987; Wang and Ker 1995). Since, so many biomaterials are viscoelastic, including bone, there are few instances when a quasistatic approximation is entirely appropriate. Eq. 1 or Eq. 2 may explain deformation in a long bone while an animal is standing still, but wholly fail to account for deformation when the animal is in motion due to drastically different loading regimes that occur on different timescales. Forces may also come from multiple directions which change over time as an animal changes the orientation of its bones and/or how it interacts with the environment.

Flexural stiffness (*EI*) measures the bending resistance of a structure. The variable *E* is the elastic modulus/Young's modulus for the structure's material, while *I* is the second moment of area of a beam's cross-section. For *EI* to represent reality, the material must be homogeneous, isotropic, linearly elastic, and experience small and equal deformations under compressive and tensile loads (Vogel 2013). However, many biological structures are heterogeneous, anisotropic, non-linear in the elastic portion of their stress-strain curves, and exposed to a wide range of loading regimes (Porter et al. 2007; Liu et al. 2014). For instance, indentation methods have been used to quantify regional heterogeneity in biocomposites (Bruet et al. 2008; Zimmerman et al. 2010; Kawano et al. 2016). *E* is a material property equal to stress (σ) over strain (ε), but is sometimes assumed to be a mechanical property, which incorporates structural variation within a material. *E* is typically quantified by preparing the material into a standard shape (e.g., dog-bone) to remove the effects of geometry. When the anatomy is important to consider, some studies apply a fixed amount of stress to a whole element (e.g., an unaltered long bone) and estimate its flexural stiffness (*EI*) based on the resulting amount of deformation (strain) or by measuring the amount of stress needed to reach a predetermined amount of strain (Horton and Summers 2009; Wilson et al. 2009; Main et al. 2010). Sometimes, comparisons of flexural stiffness can be simplified when structures are made of the same material (e.g., bone), and *E* is shared across samples. In these cases, it is the structural descriptor (second moment of area) that ultimately influences the differences in resistance to bending.

**Fig. 1:**
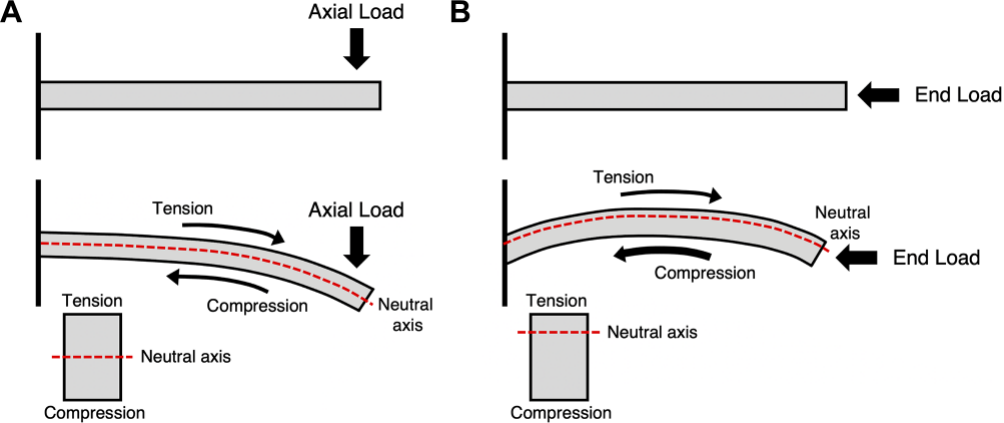
Two cantilever beams that are fixed on one end and are loaded from the side (A) and loaded at the end (B). The beam that is loaded in axial bending is in tension on top and in compression on the bottom, and experiences no stress in the middle along the neutral axis. By contrast, a larger portion of the end loaded beam is in compression than in tension and as a result, the neutral axis has shifted away from the center of mass.

Second moment of area is a measure of how the material in a beam's cross-section is arranged to resist bending and can be calculated as:

(3) \begin{equation*}{\rm{\ }}{I_{NA}} = \int {x^2}dA,\end{equation*}

where the neutral axis (*NA*) is a line perpendicular to the axis of applied force, and *x* is the distance between the neutral axis and an infinitesimal area *dA*. A beam that is loaded in axial bending develops tension on one side and compression on the other side, and the transition between these two regions experiences no stress at all (Fig. 1A). This plane of neutral (zero) stress is the neutral axis and, for beams that are not end loaded, passes through the center of mass of the cross-section. The neutral axis can provide information about a particular loading regime because it is the load that determines the orientation and position of the neutral axis (Lieberman et al. 2004). If a beam is end loaded, the neutral axis moves away from the center of mass and towards the side that is in tension (Fig. 1B). For example, the radius of a running goat has its anterior surface in compression and the posterior surface in tension at the start of stance (i.e., when the hoof initially contacts the ground). The ground reaction forces produced during the middle of stance then loads the distal end of the radius in compression, causing the lateral surface to be in tension and the medial surface to be in compression. The neutral axis rotates and shifts towards the tensile surface of the bone, the radius’ lateral surface (Main and Biewener 2004). If compression is high enough, the neutral axis no longer stays within the bone and the entire element is loaded in compression. It is worth mentioning that resistance to torsion can be measured with an analogous approach through the polar moment of area, which involves calculating the second moment of area as the squared distance of area from the center of mass (Vogel 2013).

**Fig. 2:**
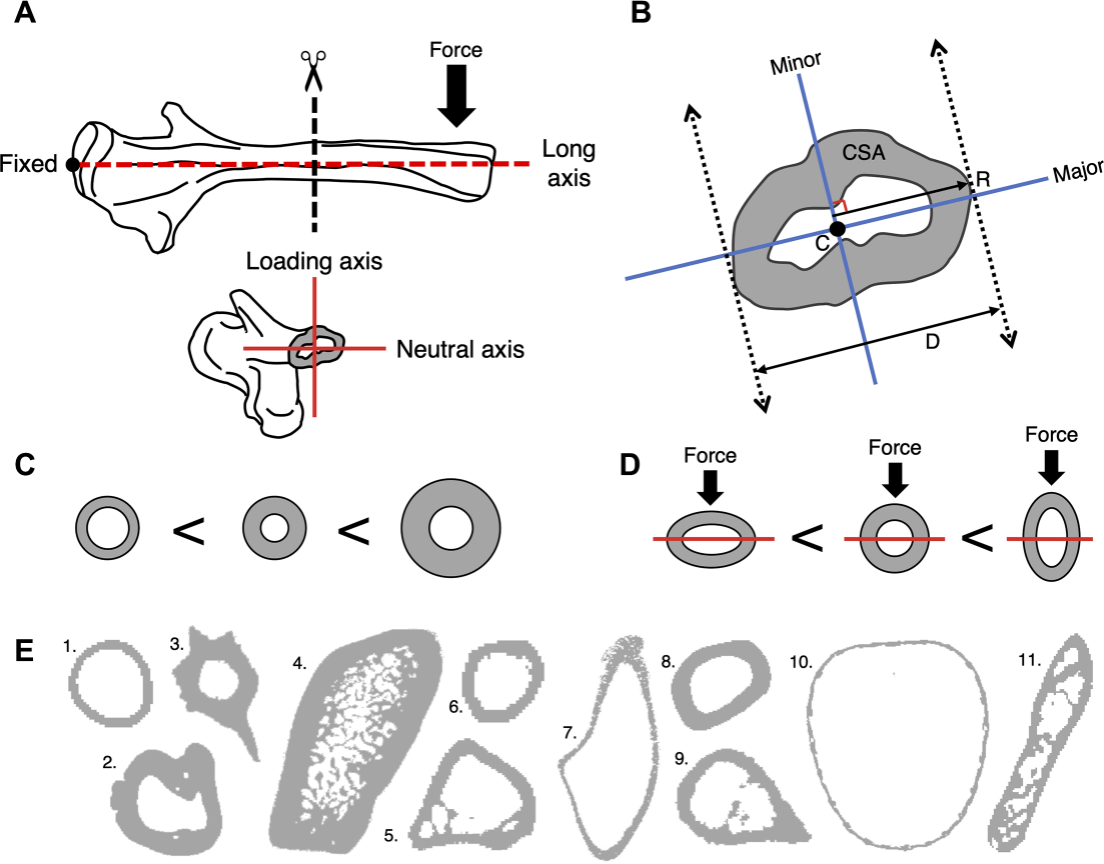
(A) A bone in a cantilevered loading regime, with a cross-section taken at mid-shaft to show the orientation of the loading and neutral axes. (B) Examples of measurements taken from the cross-sectional geometries include the CSA - cross-sectional area of the shaded region, C - centroid, R - distance to the furthest point from the minor axis, D - maximum Feret diameter. (C) Different cross-sectional shapes ranked from lowest to highest second moment of area. (D) Different cross-sectional shapes ranked from lowest to highest second moment of area when force is applied along the dorsoventral axis. (E) Example cross-sections found in nature (not to scale) - 1. Frog femur, 2. Gharial lower jaw, 3. Catfish pectoral spine, 4. Sea otter radius, 5. Chameleon humerus, 6. Bird femur, 7. Horn shark lower jaw, 8. Bat humerus, 9. Chipmunk humerus, 10. Gar fish body cavity, 11. Salamander humerus.

Quantifying the second moment of area is difficult for two reasons. First, biological structures have irregular shapes, so closed form expressions of second moment of area for canonical geometries will not capture them accurately (Fig. 2E). Computation of second moment must rely on summing the contributions of discrete area elements within the structure. Proprietary software (e.g., SolidWorks) and legacy code in MATLAB can do this but are inaccessible to most users. These calculations are not algorithmically challenging, but only two open-access tools incorporate them. The BoneJ plug-in for FIJI (Doube et al. 2010; Domander et al. 2021) is the most commonly used, but more recently, *morphomap* was developed to perform many of the same functions in an R environment (Profico et al. 2021). The *Slice Geometry* function in BoneJ iterates through a series of 2D cross-sections to calculate several geometric properties (i.e., cross-sectional area, Feret diameter, second moment of area, section modulus, etc.) and can be used on a wide variety of shapes (Doube et al. 2010; Domander et al. 2021). BoneJ also enables the user to calculate the second moment of area arbitrarily around the major and minor principal axes or define the orientation of the neutral axis when the direction of load is known or can be assumed.

A second limitation to quantifying bending mechanics in diverse structures is that traditional methods require structures to be physically sliced to visualize the internal morphology. Computed tomography (CT) scanning has become a popular method of investigating cross-sectional geometry because it is non-destructive, high-resolution, and works on hard tissue (e.g., skeletal) and soft tissue (with contrast staining). This method can be used to non-invasively slice through biological structures at any angle, which is beneficial for *in vivo* human biomechanics, rare specimens, fossils, or other specimens where dissection destroys biological context. Over the past decade there has been a transformative change in the public availability of CT scan data (Goswami 2015; Boyer et al. 2016; Davies et al. 2017; Luparell et al. 2019). Thousands of animals are now available for open-access download on MorphoSource - including all extant crocodilian species, over 200 species of sharks, and over 150 species of salamanders. However, structures must be digitally isolated and oriented before the second moment of area can be measured. Usually, the research question will require slices taken orthogonal to the long axis of a structure, but often this axis will not align with the z-axis of the image stack, which leads to incorrect results and complicates the computations. For example, an alligator mandible may be CT scanned at an angle to reduce scanning time, but without digitally aligning the anatomical axes of the mandible with the orthogonal axes of the image stack, the user would incorrectly compute second moment of area using oblique cross-sections. Neither FIJI nor R are optimized for visualizing, segmenting, transforming, or analyzing 3D CT data, so aligning the axes can be cumbersome. BoneJ can align a segmented structure with the moments of inertia, but that is not always desired. The *morphomap* R package can perform some amount of auto-segmentation and alignment but was designed specifically to analyze hominin long bones and may not be broadly applicable. Instead, many users of BoneJ or *morphomap* must perform some amount of segmentation or pre-processing in another program before beginning their analyses.

We propose that 3D Slicer (“Slicer”) (Kikinis et al. 2014), an open-source image computing platform designed for visualizing and analyzing 2D, 3D, and 4D data, is a useful tool for making these measurements. Slicer works across operating systems (Windows, MacOS, and Linux) and offers support for 3D rendering, data transformations, several manual and semi-automatic segmentation tools, linear measurements, graphical data visualization options, and a built-in Python3 environment. Functions that are not included in the core application can be developed by users and uploaded to the application's extension manager, where there are over 150 extensions currently available for download. Slicer also has its own extensive documentation, an active online forum, and a supportive user and developer community that makes it more accessible. Among organismal biologists, Slicer has recently gained traction with the help of published workflows, free workshops, and the creation of the SlicerMorph toolkit, an extension that increases Slicer's functionality for conducting 3D morphometric analyses (Buser et al. 2020; Porto et al. 2021; Rolfe et al. 2021). For these reasons, Slicer provides an ideal platform to streamline workflows by implementing the capabilities to measure second moment of area and other metrics of cross-sectional geometry.

Here, we present *SegmentGeometry*, a new extension for Slicer that is designed to serially calculate the second moment of area and other cross-sectional properties along the length of 3D structures. Below, we describe the functionality of *SegmentGeometry* and how the second moment of area is calculated. We present two use cases to demonstrate the utility of *SegmentGeometry* and highlight the interpretations of second moment of area in diverse biological structures.

## SegmentGeometry

*SegmentGeometry* is a Python-based extension for Slicer developed to run on the current stable release of Slicer (v4.11, r29738). The official method of installing *SegmentGeometry* and its dependency, *SegmentEditorExtraEffects*, is through Slicer's built-in extension manager. Detailed documentation about *SegmentGeometry*, step-by-step instructions on how to install and use *SegmentGeometry*, demonstration videos, and the source code are all provided on a GitHub repository (https://github.com/jmhuie/Slicer-SegmentGeometry).

Because vertebrate CT scans currently dominate public repositories, we anticipate most use cases will involve the analysis of skeletal material. However, the utility of *SegmentGeometry* extends beyond vertebrates. This tool can be used to process plants, invertebrates, and even non-CT data, as long as a series of cross-sectional images is provided. *SegmentGeometry* integrates with other Slicer extensions and modules to form a powerful platform for analyzing a diversity of use cases. For example, the *Transforms* module in Slicer provides a simple way to orient the long axis of a structure with the z-axis, allowing the user to slice through an arbitrarily defined axis. *SegmentGeometry* presents a set of interactive tools for the manual rotation of segments, and like BoneJ, automatic alignment of a segment's principal axes (based on the segment's moments of inertia) with the xyz-axes of the image stack. *SegmentGeometry* will also plot the second moment of area along the length of the structure for quick visualization and generate a table that can be imported into statistical analysis software.

### Second moment of area

Canonical shapes have equations for calculating their second moment of area, while those of non-canonical polygons can sometimes be calculated by breaking them down into simpler shapes and summing the second moment of area of their parts. Both *SegmentGeometry* and BoneJ have broad applicability because they can analyze arbitrary shapes by finding the second moment of area of each rectangular pixel and summing them to find the total for the shape. Both programs use the parallel axis theorem, which makes it easier to calculate the second moment of area of a pixel that does not fall along the neutral axis. The second moment of area of an arbitrary composite shape around the neutral axis (NA) is calculated as:

(4) \begin{equation*}{I_{NA}}\ = \mathop \sum \limits_{k\ = \ 1}^n {I_{N{A^{\prime}}k}} + {A_k}{D_k},\ \end{equation*}

where *NA’* is an arbitrary axis that crosses pixel *k* and is parallel to the neutral axis, *I_NA'k_* is the second moment of area of the pixel around the arbitrary axis, *A_k_* is the area of the pixel, *D_k_* is the perpendicular distance between the arbitrary axis and the neutral axis, and *n* is the number of pixels in the cross-section.

*SegmentGeometry* follows BoneJ in implementing second moment of area calculations around the major and minor principal axes, and a user-determined neutral axis (Doube et al. 2009, 2010; Domander et al. 2021). The principal axes are defined as two perpendicular lines that intersect at the centroid and are orientated so the product moment of inertia equals zero. The second moment of area around the minor principal axis (*I_minor_*) and major principal axis (*I_major_*) represent the highest and lowest bending resistance for a given cross-section, respectively. The user may define the orientation of their own centroidal neutral axis using the interactive interface in *SegmentGeometry.* When the option to use a custom neutral axis is selected, a line will be drawn, which can be rotated by the user to represent the angle of the desired neutral axis relative to the horizontal. Alternatively, the user may provide an exact angle to define the orientation of the neutral axis. Unlike the principal axes, where the orientation will vary from slice to slice, the angle of the user-determined neutral axis is used for the entire length of the structure.

To compare the second moment of area between differently sized structures, it may be useful to normalize the values. The second moment of area can increase through three main mechanisms: increasing the size of the cross-section, investing more material into the section, or moving the material away from the center and towards the direction of loading (Fig. 2C and 2D). To help control for these potential sources of variation, *SegmentGeometry* implements two methods of normalization, one that normalizes by the length of the beam and the other that normalizes by the amount of material in the cross-section. The length normalization takes the second moment of area, which has a unit of mm^4^, and reduces it to a linear measurement by taking the fourth root and then divides it by the length of the whole structure (Doube et al. 2009). This method ameliorates the effects of beam length and enables the comparison of structures that vary in size, attributing variation in second moment to changes in shape, material investment, and cross-section size. In some cases, correcting for beam length will also ameliorate the effects of cross-section size if there is an isometric relationship. To help isolate the effects of shape, the material normalization divides the second moment of area measured from the structure by the second moment of area of a solid circle with the same cross-sectional area (Summers et al. 2004). The result is a ratio that quantifies how much better or worse the material in a structure is arranged to resist bending than a solid, circular cylinder.

### Additional parameters

*SegmentGeometry* will output additional cross-sectional properties such as the cross-sectional area, maximum Feret diameter, perimeter, average pixel brightness, angle of the principal axes, section modulus, and polar moment of inertia. Section modulus quantifies the overall bending strength of a beam's cross-section and is calculated as the second moment of area divided by the perpendicular distance to the furthest pixel away from the neutral axis. If the structure were to bend, that pixel is where the structure would bend first. Polar moment of inertia represents a beam's ability to resist torsion based on its cross-sectional shape. It is calculated as the sum of the squared distance between the pixel and the centroid multiplied by the pixel's area, for each pixel in the cross-section. Cross-sectional area (mm^2^), section modulus (mm^3^), and polar moment of inertia (mm^4^) can be size-corrected through the length normalization procedure; however, the respective roots are taken to reduce them to linear values. Section modulus and polar moment of inertia can also be corrected through material normalization by finding the ratio between the structure's measured values and the estimated values for a solid circle with the same cross-sectional area. A third normalization procedure specific to cross-sectional area measures material compactness, or the cross-sectional area of the structure in a given slice divided by the area plus any vacuities within the section. Compactness is generally used in the context of bones to compare differences in material investment. *SegmentGeometry* will use the smallest calculated maximum Feret diameter, ignoring the first 5% of the structure on both ends to account for variability, and the length of the structure to determine the aspect ratio of the beam. If the length to width ratio is under 10, there will be a warning that notifies the user that the no-shear assumption of the Euler-Bernoulli beam theory may not be met.

### Limitations

*SegmentGeometry* has the benefit of being open-access, open-source, user-friendly, and broadly applicable to a wide range of uses, but does have caveats that users should consider. First, users are restricted to calculating the second moment of area around a neutral axis that passes through the centroid, but that condition may not be appropriate for all use cases. For instance, the neutral axis does not pass through the centroid if a beam is end loaded, which is important for bones that are weight bearing. Continued development of *SegmentGeometry* could enable second moment of area calculations around non-centroidal axes, but *in vivo* experimental data (which are rare) are needed to calculate absolute results. Using centroidal axes may not capture real-world differences in stiffness, but it is still appropriate for making relative comparisons between structures and drawing pattern-based conclusions (Lieberman et al., 2004). Second, the distribution of mineral density can affect the geometry of a structure and where the neutral axis lies. The center of mass is at the geometric center in a cross-section with homogeneous distribution of material but will deviate if material density is unevenly distributed across the section. Density-weighted calculations may provide more realistic numbers to ameliorate this problem, but comparing density measurements between CT scans is fraught with errors. It requires a standardization of grayscale values or the use of phantoms, objects of known density, to translate pixel brightness into mineral density values. Most CT scans in online repositories do not fall into either category, making density-weighted measurements a very specific use case. Lastly, *SegmentGeometry* provides multiple normalization features that allow users to investigate the factors that affect second moment of area at greater detail, but currently no method is provided to isolate or account for cross-section size. That is in part because choosing a proxy for cross-section size (i.e., width, area, perimeter, etc.) is difficult when the shape of the cross-sections are highly variable. Nevertheless, the beauty of open-source software is that any of these features have the possibility of being realized in the future.

## Use Cases

### Use Case #1: Assessing the direction of bending in the salamander humerus

**Fig. 3:**
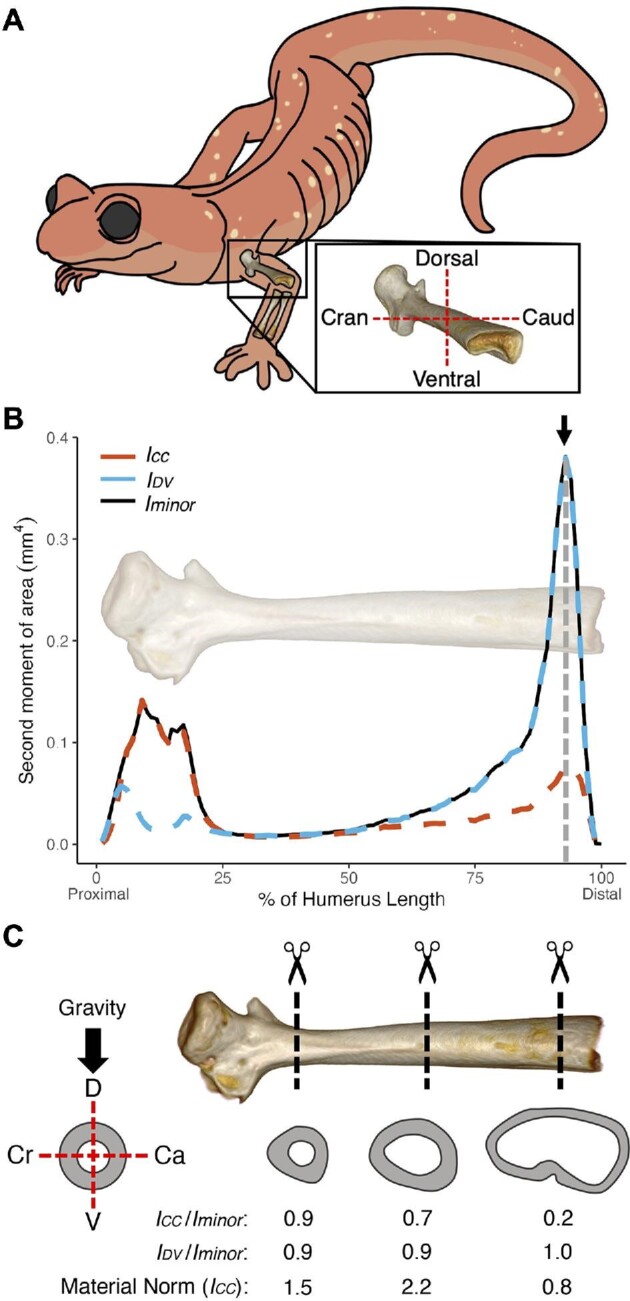
Variation in the second moment of area across the humerus of an *Aneides lugubris* salamander. (A) The orientation of the humerus while the animal is mid-stance and how the bone was oriented in 3D Slicer. (B) Second moment of area varies along the length of the bone. The arrow indicates a point on the bone where there is high directionality; specifically, bending resistance around the dorsoventral axis is nearly 4 times higher than the bending resistance around the craniocaudal axis (recall that the direction of force is perpendicular to the axis around which bending occurs). (C) Three example cross-sections. The first two rows of numbers report the correspondence in bending resistance of each cross-section about the craniocaudal (*I_CC_*) and dorsoventral (*I_DV_*) axes relative to the highest bending resistance about the minor principal axis (*I_minor_*). The third row reports material normalized *I_CC_* values.

Bones are loaded in distinct directions during locomotion, which can be mapped on to the anatomy of the long bones to assess form-function relationships. One hypothesis is that the midshaft of a long bone (e.g., humerus) should have the greatest stiffness in the direction where the largest loads are applied to the bone (Lieberman et al. 2004). Salamanders are sprawling quadrupedal amphibians that hold their upper limbs horizontal relative to their body and parallel to ground (Fig. 3A). Empirical data from force plate experiments have demonstrated that the ground reaction forces imposed on individual forelimbs during terrestrial locomotion are predominantly oriented in the vertical direction and slightly caudally for the semi-aquatic *Pleurodeles waltl* and terrestrial *Ambystoma tigrinum* (Kawano and Blob 2013, 2021). Therefore, the shape of the salamander forelimb may reflect its loading regime and be stiffer in these directions. Bone loading analyses and second moment of area calculations on the humeri of the terrestrial *A. tigrinum* confirmed that resistance to bending was greatest in the dorsocaudal direction, reflecting the directionality of the ground reaction forces (Kawano et al. 2016). However, it is unclear whether these patterns are found in other terrestrial salamanders and whether there is variation along the length of a limb bone. To demonstrate the power of *SegmentGeometry* and investigate these patterns, we downloaded a micro-CT scan of the arboreal salamander (*Aneides lugubris*), a terrestrial plethodontid species, from MorphoSource (http://n2t.net/ark:/87602/m4/M49486). We loaded the scan into 3D Slicer with the SlicerMorph extension and segmented the left humerus with the *Segment Editor* module. We used the rotation tools in *SegmentGeometry* to orient the humerus to mimic its orientation when the animal is mid-stance, associating the horizontal and vertical axes of the bone's cross-sections with the craniocaudal (CC) and dorsoventral (DV) axes of the animal's body, respectively (Fig. 3A). *SegmentGeometry* was used to calculate the second moment of area around (1) the CC axis, (2) the DV axis, and (3) the minor principal axis (the axis about which bending resistance is expected to be the highest), along the length of the bone. The material normalization method was applied to the second moment of area values.

The second moment of area and, therefore, the resistance to bending varied along the length of the humerus for *A. lugubris* (Fig. 3B). The values suggest that bending resistance near the epiphyses is relatively greater than regions in the diaphysis. There was also substantial directional anisotropy in bending mechanics near the epiphyses. For example, near the distal end of the humerus, the second moment of area suggests that the bending resistance to loads applied along the craniocaudal axis is four times higher than the bending resistance to loads applied in the dorsoventral direction (indicated by the arrow in Fig. 3B). In contrast, at the proximal end of the humerus, the bending resistance to dorsoventral loads is the highest. The close correspondence between the minor principal axes and the anatomical axes (calculated as the ratio of their second moments of area) suggests the humerus is shaped to resist predictable loads acting very near the anatomical axes (Fig. 3B and C). Lastly, the material normalization can give us insights into how well a particular element is designed to withstand flexion. Considering the midshaft of the bone, the ratio between the second moment of area of the bone about the craniocaudal axis and the second moment of area of a circle with the same cross-sectional area shows that the mineral is arranged to be 2.2 times better at resisting dorsoventral loads than if it were a solid, circular beam. Expanding such calculations across a broader sampling of taxa would help clarify how resistance to bending varies across salamanders with different body sizes and ecologies.

### Use Case #2: Comparing jaw stiffness in durophagous sharks

Sharks are a group of cartilaginous fishes that feed on a wide range of prey items, and some specialize in crushing and eating hard-shell prey items (*Heterodontus spp.* and *Sphyrna tiburo*). Cartilaginous durophages are surprising because their jaws are softer than the prey they are crushing. However, elasmobranchs cover their skeleton in a thin layer of calcified tiles (called “tesserae”) that increase its stiffness, and have a pavement of teeth. The lower jaw of durophagous sharks has higher bending resistance in the posterior region, where crushing is performed, relative to the anterior region (Summers et al. 2004; Herbert and Motta 2018). However, the morphology of these species has only been examined in isolation, so here we ask whether durophagous sharks have exceptionally stiff jaws compared to other sharks. We downloaded micro-CT scans for 10 species of sharks from MorphoSource and the #ScanAllFish OSF project (https://osf.io/ecmz4/), including the durophagous Mexican horn shark (*H. mexicanus*) and bonnethead (*S. tiburo*) (Table S1). For each specimen, we segmented the left side of the lower jaw in Slicer and oriented the jaws so the direction of crushing force was perpendicular to the cross-sections’ horizontal axis. The crushing axis was inferred from the occlusal surface between the upper and lower jaw. We used *SegmentGeometry* to slice through the long axis of the jaws from the symphysis to the jaw joint, and calculate the second moment of area around the horizontal axis. We applied both the length and material normalization to the second moment of area values and calculated jaw compactness (cross-sectional area divided by total area).

**Fig. 4:**
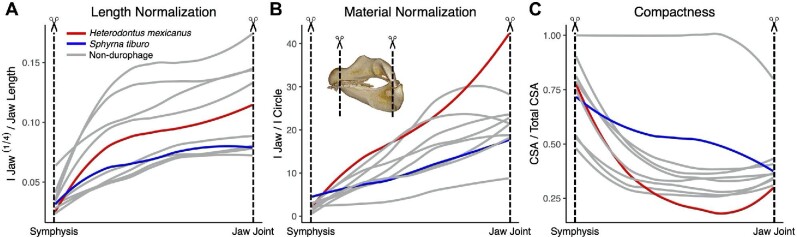
The second moment of area of the lower jaw about the axis perpendicular to the direction of crushing forces in ten shark species between the symphysis and the jaw joint. (A) The length normalized values show that for the size of their jaws, the durophagous sharks have average bending resistance relative to other sharks. (B) The material normalized values show that the shape of the *Heterodontus* jaw (red) is more efficient for resisting bending than non-durophagous sharks (grey), while the jaw of *Sphyrna* (blue) is not. (C) A measure of compactness shows that *Sphyrna* increases its jaw stiffness by investing more material, but that *Heterodontus* invests relatively little compared to other sharks.

The length and material normalizations tell two different stories (Fig. 4A and B). In both cases, the ability of the jaw to resist crushing forces increased in the anterior to posterior direction for all sharks. However, the material normalization indicated that the lower jaw of *H. mexicanus* had one of the most efficient structural designs compared to other sharks, while *S. tiburo* had a below average design for resisting bending (Fig. 4B). In contrast, the length normalization indicated that for the length of their jaws (distance between the symphysis and the jaw joint), both durophagous sharks are simply average at resisting bending (Fig. 4A). The length normalization differs from the material normalization in that it accounts for differences in the length of the shark jaws but not any of the mechanisms that influence the second moment of area (e.g., cross-section size, shape, and material investment). The material normalization corrects for both the size of the cross-sections and material investment, isolating the effects of shape. When comparing differences in compactness, which represents how much material is invested in the lower jaw while accounting for cross-section size, *H. mexicanus* has less tesserae in its jaw than most sharks, while *S. tiburo* has above average (Fig. 4C). *Heterodontus mexicanus* has invested less calcified material in its jaw compared to other sharks, but it arranges the material that it does have to optimize resistance to crushing forces. Meanwhile, *S. tiburo* has a worse jaw design but compensates by investing more material into its jaw. Thus, the two durophages have adopted different strategies for building stiff jaws that can resist the forces of crushing hard-shell prey.

## Conclusions

With the increase in the number of publicly available CT scans, there is a real need for open-access, open-source software that can process hundreds of specimens in a short time frame. We developed *SegmentGeometry*, an extension for 3D Slicer, to iterate slice-by-slice along the length of a 3D structure and analyze its cross-sectional geometry. Second moment of area, as defined by the Euler–Bernoulli beam theory, is a powerful tool for studying the relationship between structure and bending resistance, which has already been applied to a diverse range of biological structures. Second moment of area is useful for identifying the direction of bending in a beam in relation to loading regimes (e.g., a salamander humerus), and provides the means to compare ecologically diverse taxa on a macroevolutionary scale (e.g., shark jaws). However, we have only scratched at the surface on the kinds of questions that can be answered with second moment of area data and the rest of the beam theory equation. We hope that *SegmentGeometry* becomes a useful tool for organismal biologists to analyze the mechanical properties of biological structures.

## Supplementary Material

IOB_obac009_Supplemental_Materials

## Data Availability

The data collected for this study are available in the supplemental materials. Links to the micro-CT scans used in this study are provided in the article or supplemental materials.
